# Status of mental and social activities of young and middle-aged patients after papillary thyroid cancer surgery

**DOI:** 10.3389/fonc.2024.1338216

**Published:** 2024-03-26

**Authors:** Songhao Chen, Xi’e Hu, Ping Yang, Lin Yang, Shujia Peng, Li He, Lijuan Yuan, Guoqiang Bao

**Affiliations:** ^1^ Department of General Surgery, The Second Affiliated Hospital of Air Force Medical University, Xi’an, China; ^2^ Department of Obstetrics and Gynecology, The 920 Hospital of Joint Logistic Support Force of Chinese People’s Liberation Army (PLA), Kunming, China; ^3^ Department of Pathology, The University of Oklahoma Health Sciences Center, Oklahoma City, OK, United States

**Keywords:** papillary thyroid cancer, quality of life, mental state, social activities, young and middle-aged

## Abstract

**Background:**

Papillary thyroid cancer (PTC) is prevalent among younger populations and has a favorable survival rate. However, a significant number of patients experience psychosocial stress and a reduced quality of life (QoL) after surgical treatment. Therefore, comprehensive evaluations of the patients are essential to improve their recovery.

**Methods:**

The present study enrolled 512 young and middle-aged patients diagnosed with PTC who underwent surgery at our institution between September 2020 and August 2021. Each participant completed a series of questionnaires: Generalized Anxiety Disorder 7 (GAD-7), European Organization for Research and Treatment of Cancer Quality of Life Questionnaire (EORTC QLQ-C30), Thyroid Cancer-Specific Quality of Life Questionnaire (THYCA-QoL), and Readiness to Return-to-Work Scale (RRTW).

**Results:**

GAD-7 data showed that almost half of the study subjects were experiencing anxiety. Regarding health-related quality of life (HRQoL), participants reported the highest levels of fatigue, insomnia, voice problems, and scarring, with patients in anxious states reporting worse symptoms. Based on RRTW, more than half of the subjects had returned to work and had better HRQoL compared to the others who were evaluating a possible return to work. Age, gender, BMI, education, diet, residence, health insurance, months since surgery, monthly income, and caregiver status were significantly correlated with return to work. Additionally, having a caregiver, higher monthly income, more time since surgery, and living in a city or village were positively associated with return to work.

**Conclusion:**

Young and middle-aged patients with PTC commonly experience a range of health-related issues and disease-specific symptoms following surgery, accompanied by inferior psychological well-being, HRQoL, and work readiness. It is crucial to prioritize timely interventions targeting postoperative psychological support, HRQoL improvement, and the restoration of working ability in PTC patients.

## 1 Introduction

Thyroid cancer (TC) has become the most prevalent malignancy of the endocrine system worldwide in the last few decades (1). Females make up the majority of TC patients (2, 3), and papillary thyroid cancer (PTC) is the most predominant type of TC, accounting for 95% of differentiated thyroid cancers (4, 5). In China, the incidence of TC has increased dramatically in the last 30 years, and the National Cancer Center of China reported that approximately 220,000 new cases of TC were diagnosed in 2022, with the disease tending to develop at a younger age (6, 7). Surgery is the most common treatment for TC, and the 10-year overall survival rate for patients can reach 90% or higher (8, 9). However, a significant proportion of patients suffer from postoperative sequelae that have serious repercussions for their quality of life (QoL), such as anxiety and depression, voice changes, and scarring (10, 11), which can negatively impact both their mental and physical recovery (12, 13). Therefore, given the high postoperative survival rate for patients with PTC, it is essential to consider health-related quality of life (HRQoL) and social function when assessing the recovery quality of patients after surgical procedures.

HRQoL is now considered an essential measure to assess the outcomes of clinical treatments and interventions (14, 15). Although it is widely acknowledged that PTC has a better clinical cure rate and lower death rate than other malignant diseases (16–18), the HRQoL status of postoperative patients with PTC is similar to or even lower than that of patients with other malignant tumors (19–21). Therefore, timely targeted intervention measures after surgical treatment in PTC patients have become an increasingly important goal (22). Improving social activity function and HRQoL (23) is crucial in achieving this goal. However, few studies have been performed to assess the relationship between HRQoL and social function in postoperative PTC patients as well as their potential influencing factors.

In addition, there remains a lack of comprehensive studies on the postoperative psychological status, social activity ability, and QoL in these patients. A study conducted by Wang et al. in Hangzhou, China examined HRQoL in community-based survivors of thyroid cancer and included a large sample size as well as an exploration of multiple factors associated with QoL (24). However, the study did not investigate the psychological status and social activities of the patients, and the clinical information was collected from individuals who had experienced thyroid cancer many years ago, leading to potential recall bias. Another recent study focused on investigating the psychological status of PTC patients who underwent surgery but similarly lacked a comprehensive examination of other postoperative aspects (25). Similarly, several other studies primarily concentrate on evaluating either postoperative QoL (26–29) or solely assessing the postoperative psychological well-being of patients (30–33), while neglecting other important dimensions. Therefore, conducting comprehensive studies encompassing analysis across multiple dimensions and their related factors in PTC patients after surgery is still necessary.

This study aims to comprehensively evaluate the psychosocial performance of patients with PTC after surgery, including their psychological state, QoL, readiness to return to work, and their related factors. The findings from this study may provide clinical guidance for individualized interventions in patients with PTC postoperatively, with the ultimate goal of improving their QoL after surgery.

## 2 Methods

### 2.1 Setting and population

This study is a population-based cross-sectional survey conducted at the Second Affiliated Hospital of Air Force Medical University, Xi’an, China, from September 1st, 2020, to August 31st, 2021. Patients who had pathologically diagnosed PTC according to the American Joint Committee on Cancer (AJCC) 8th edition guidelines (9) were recruited. The Ethics Committee for Clinical Research and Animal Testing of the Second Affiliated Hospital of Air Force Medical University approved this cross-sectional study (ethical approval number: K202205-20), and all patients provided written informed consent. No financial compensation was provided to the patients.

The inclusion criteria for participants were as follows: (a) aged between 18 and 59 years (defined as middle-aged); (b) had undergone surgical procedures and been diagnosed with PTC through postoperative pathology; (c) were conscious, had normal hearing, and had normal cognitive comprehension and expression abilities without any psychiatric disorders; and (d) agreed to participate in the study and signed an informed consent form.

The exclusion criteria for this study were patients who (a) were pregnant; (b) experienced serious postoperative complications (e.g. nerve injury and iatrogenic hypoparathyroidism); (c) had a history of anxiety, depression, or organic psychosis; (d) had undergone surgery for tumors in the past; (e) had received radioactive iodine treatment; and (f) had primary hyperthyroidism or hypothyroidism before surgery.

### 2.2 Data collection

The relevant information of the patients was collected in 1-6 months after surgery in this study. The basic information, including sociodemographic and clinical characteristics, of all eligible patients, was collected through self-report and hospital case records. Specifically, the data included age, gender, ethnicity, educational background, smoking and drinking habits, dietary status, residence, marital status, health insurance, time after surgery, occupation, monthly income, caregiver, type and scope of surgery, intake of levothyroxine, the volume of physical activity per week, and daily intake of fruits and vegetables. Notably, the participants’ weekly physical activity was measured as the number of days per week that they performed moderate-intensity exercise for 30 minutes (e.g., brisk walking and housework) (34), and responses were scored on a four-point scale: 1 = 0 days; 2 = 1 to 2 days; 3 = 3 to 4 days; 4 = minimum 5 days. Moreover, the intake of 100 g of raw fruits and vegetables was considered one portion, and participants reported their intake (24, 35).

Importantly, all eligible patients were asked to complete four standard questionnaires that have been widely used and have demonstrated robust reliability and validity in Chinese, including Generalized Anxiety Disorder (GAD-7) (36, 37), European Organization for Research and Treatment of Cancer Quality of Life Questionnaire (EORTC QLQ-C30) (38–40), Thyroid Cancer-Specific Quality of Life Questionnaire (THYCA-QoL) (8, 41) and Readiness to Return-to-Work Scale (RRTW) (42).

The GAD-7 questionnaire was used to evaluate potential postoperative anxiety. The main statistical indicator of the GAD-7 is the total score, which ranges from 0 to 21 and is the sum of the item scores. The severity of anxiety can be assessed based on the GAD-7 scores: a score of 0-4 indicates no anxiety, 5-9 indicates mild anxiety, 10-14 indicates moderate anxiety, and 15-21 indicates severe anxiety. Notably, the GAD-7 is also used as a diagnostic tool for anxiety symptoms, with a cutoff value of 10 or higher (43).

Additionally, the EORTC QLQ-C30 was used to investigate the general profile of QoL in this study. It contains 30 items, including a global QoL scale, five functional scales (physical, role, cognitive, emotional, and social), three symptom scales (fatigue, pain, nausea, and vomiting), and several single items assessing common symptoms (dyspnea, loss of appetite, insomnia, constipation, diarrhea, and financial difficulties). The questions referred to the previous week, and each item was scored on a four-point response scale ranging from 1 = “not at all” to 4 = “very much,” except for QoL, which was scored on a seven-point modified linear analog scale ranging from 1 = “very poor” to 7 = “excellent.” After linear transformation, all scales and individual items were measured from 0 to 100. Higher scores on the functional scale and QoL indicate better functioning and HRQoL, while higher scores on the symptom scale indicate more complaints.

In addition to the EORTC QLQ-C30, the THYCA-QoL was also utilized in our study to evaluate the QoL of PTC patients postoperatively. The THYCA-QoL scale is a thyroid cancer-specific QoL scale designed to measure specific issues in patients with TC. It is used along with the Chinese version of the EORTC QLQ-C30 to assess the overall impact of TC and treatment on patients’ QoL. The scale consists of 24 items divided into seven symptom scales (neuromuscular, voice, concentration, sympathetic, throat/mouth, psychological, and sensory) and six single items (scar, chilly, tingling, weight gain, headache, and sex). All items refer to the past week, except for the sexual interest item, which refers to the past 4 weeks. All items were rated on a 4-point scale (not at all, somewhat, fairly, and very much) and scored from 1 to 4. The scores for each scale are calculated in the same way as for the EORTC QLQ-C30.

Of note, to examine the employment status of patients with PTC after surgery, we used the RRTW scale. The RRTW scale is a 22-item questionnaire that consists of different components for working (9 items) and nonworking individuals (13 items). For working individuals, there are two stages: uncertain maintenance (UM), which explores the worker’s struggle to remain employed, and proactive maintenance (PM), which examines the coping mechanisms and strategies to manage work in difficult situations that could lead to a setback. For the nonworking population, there are four stages. The precontemplation (PC) stage documents the lack of desire or plan to return to work. The contemplation (C) stage refers to when the individual begins to consider returning to work (RTW). The preparation for action-self-evaluative (PA-S) stage measures the degree of readiness for RTW, strategies to make work manageable, and making an actual plan for RTW (e.g., determining an actual date). Finally, the preparation for action-behavioral (PA-B) stage measures the degree of mental readiness and active involvement of the individual in building strength for RTW. The RRTW scale has demonstrated satisfactory construct validity for most stages of vocational rehabilitation and has shown an association with actual work outcomes.

### 2.3 Statistical analysis

Descriptive statistics were used to analyze the basic demographic data of patients with PTC after surgery, with categorical variables presented as percentages and continuous variables presented as the means and standard deviations (SD). Categorical variables were compared using either the chi-square test or Fisher’s exact test. A two-tailed p-value of <0.05 was considered statistically significant. Univariate logistic regression analysis was performed to identify relevant variables associated with anxiety and return-to-work status. Variables with a p-value of <0.2 were included in the multivariable regression model as independent predictive factors associated with psychological status and RRTW after surgery in young and middle-aged PTC patients (44). Backward stepwise selection was applied using the likelihood ratio test. All statistical analyses were performed using SPSS version 26.0 (IBM Corporation, Armonk, NY).

## 3 Results

### 3.1 Characteristics of the study population

**Figure 1:**
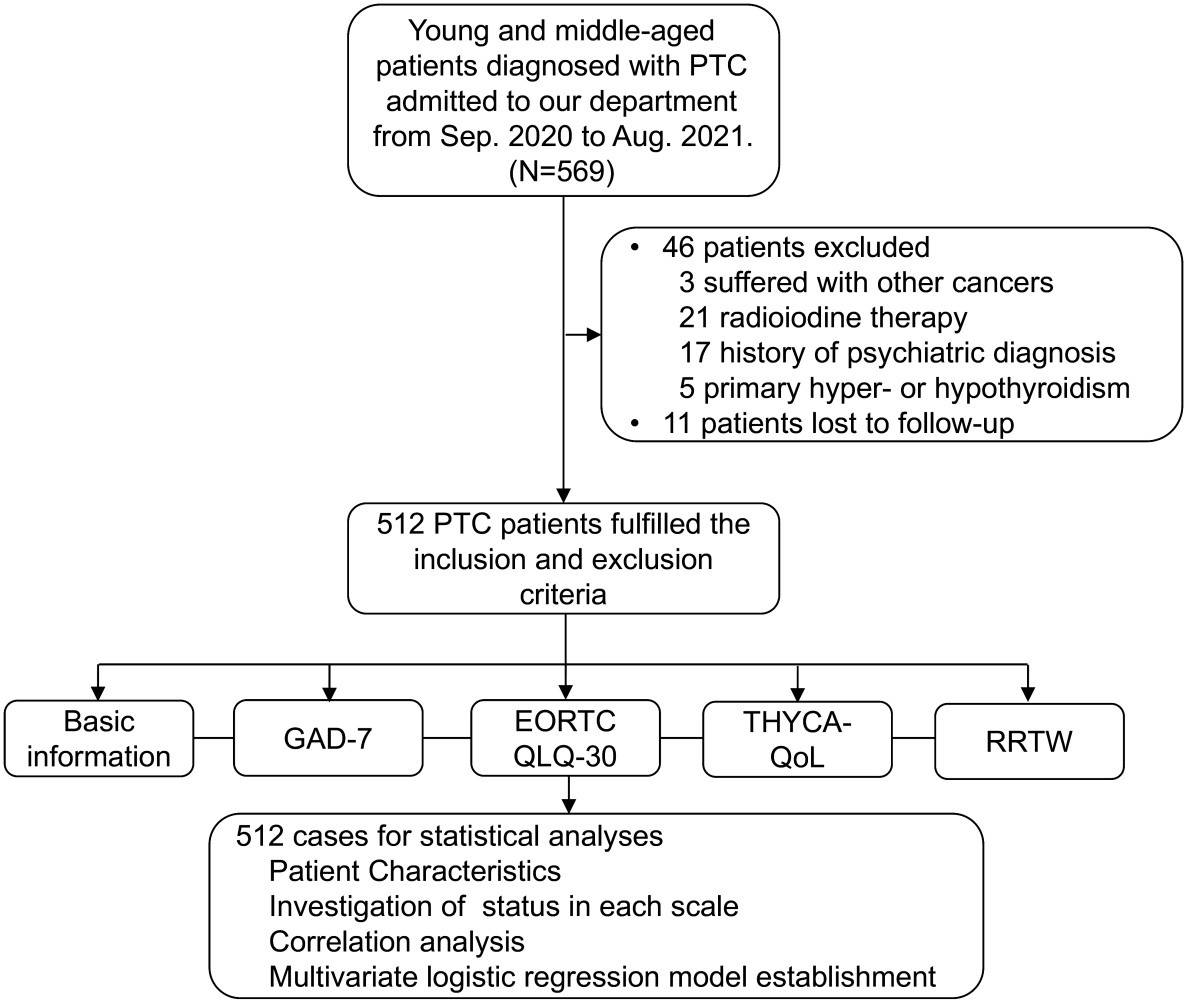
Diagram of the Data Collection Process. This study included young and middle-aged patients diagnosed with PTC admitted to our department from Sep. 2020 to Aug. 2021; a total of 569 patients were enrolled firstly. Then, according to our inclusion and exclusion criteria, 46 patients were excluded as follow, 3 patients suffered with other cancers; 21 patients radioiodine therapy; 17 patients had a history of psychiatric diagnosis; 5 patients suffered from primary hyper- or hypothyroidism; and 11 patients lost to follow-up. Finally, a total of 512 PTC patients fulfilled the criteria and were included in this study.

In this study, 569 patients were diagnosed with PTC and underwent thyroid surgery during the research period. Ultimately, 512 patients were included in the study based on the inclusion and exclusion criteria (**Figure 1**). As shown in **Supplementary Table S1** and **Supplementary Figure S1**, the PTC patients who participated in the research were predominantly female, accounting for 81.3% of all patients. The age distribution was mainly between 30 and 50 years, accounting for 59.4%. Over one-third (175/512, 34.2%) of the patients had a bachelor’s degree or higher education. The majority of the patients were nonsmokers (91%) and nonalcohol drinkers (87.5%). Additionally, the patients mostly resided in cities (68.8%), with the number of people living in rural areas and villages being almost equal (17.6% and 13.7%, respectively). Most of the patients were married (90%), and only 1.8% and 1.4% of the participants were divorced or widowed, respectively. Notably, medical health insurance covered the majority of the study population, with 502 (98.0%) patients having insurance. More than half (292/512, 57.0%) of the patients participating in the study were employees or full-time students; almost half of the participants (46.1%) received a monthly income in the range of ¥2000-5000. In terms of surgical procedure choice, 330 patients (64.5%) preferred traditional open surgery, and it should be noted that a total of 260 (50.8%) of them had undergone thyroidectomy and lymphatic dissection, which increased both the scope of practice and the complexity of the surgery. During the postoperative recovery process, the majority of patients were cared for by their relatives (358/512, 69.9%); the other 154 patients lacked personal caregivers after surgery. Furthermore, the vast majority of patients had high postoperative compliance and were able to take levothyroxine regularly (504, 98.4%). Additionally, more than one-third of the patients (196/512, 38.3%) performed 1-2 days of exercise per week during postoperative recovery. However, the overall fruit and vegetable intake of the surveyed population was very low, with only 60 patients (11.7%) consuming more than 2 portions of fruit and vegetables per day.

### 3.2 Evaluation of the anxiety status and its related factors of the study population after surgery

All patients with PTC were given a final standard score that allowed for an assessment of their anxiety level according to the GAD-7. As shown in **Table 1**, no one had a normal score, with 249 subjects experiencing mild anxiety (mean GAD-7 score of 7.49 ± 0.73), 179 experiencing moderate anxiety (mean GAD-7 score of 12.20 ± 1.40), and 84 suffering from severe anxiety (mean GAD-7 score of 17.94 ± 2.48). The cutoff value for anxiety when using GAD-7 as an auxiliary diagnosis is 10 (i.e., a GAD-7 score ≥10 is considered anxiety clinically). Based on this cutoff value, the study population was divided into two groups: nonanxiety (249 individuals, 48.63%) and anxiety (263 individuals, 51.37%).

**Table 1:** Investigation of the anxiety status according to GAD-7 in PTC survivors (N = 512).

Anxiety degree	Score range	n	%	GAD-7 score (M ± SD)
**No**	0-4	0	0	0
**Mild**	5-9	249	48.63	7.49 ± 0.73
**Moderate**	10-14	179	34.96	12.20 ± 1.40
**Severe**	15-21	84	16.40	17.94 ± 2.48

GAD-7, the Generalized Anxiety Disorder; PTC, papillary thyroid cancer; M, mean scores; SD, standard deviation.

To explore the factors associated with anxiety, we conducted a comparative analysis of various characteristics between patients with PTC who were and were not experiencing anxiety. The data demonstrated a significant difference in age between the two groups (*P* = 0.042), indicating that patients with PTC aged between 31 and 40 were more likely to experience anxiety after receiving thyroid surgery compared to other age groups. Moreover, there was a statistically significant difference in anxiety status between males and females. Specifically, anxiety symptoms were more prevalent in females than in males (85.9% *vs*. 14.1%, *P* = 0.005). Additionally, the daily intake of vegetables and fruits emerged as a potential indicator of anxiety among patients with PTC. Patients who consumed either too few or too many fruits and vegetables per day were more likely to experience anxiety (*P* = 0.041). In summary, our data revealed that age, gender, and daily fruit and vegetable intake were significant factors associated with anxiety status in the study subjects (**Supplementary Table S2**).

### 3.3 Evaluation of the RRTW and its related factors of the study population after surgery

In the study, 63 participants were retired or unemployed before the diagnosis of PTC. Accordingly, we finally included 449 patients for the investigation of RRTW. Our data showed that 151 patients did not return to work after surgery, while 298 patients returned to work, which is approximately twice the number of those who did not return to work.

The items on the RRTW scale ranged from 1 to 5. The results of the study showed that patients who did not return to work had a higher average score on item 11 (They wished they had more ideas about how to return to work) and item 12 (They desired advice on how to go back to work) (**Table 2**). In contrast, patients who had returned to work had a higher average score on item 14 (They were doing everything they could to stay at work) and item 21 (They had returned to work and were doing well), indicating a positive attitude toward their work (**Table 3**).

**Table 2:** RRTW scores of PTC survivors who were not back at work (N = 151).

Items of RRTW	Dimension	Scores (M ± SD)
**1. You do not think you will ever be able to go back to work.**	Precontemplation	2.47 ± 1.10
**2. As far as you’re concerned, there is no point in thinking about returning to work.**	Precontemplation	2.69 ± 1.19
**3. You are actively doing things now to get back to work.**	Prepared for action—Behavioral	3.50 ± 1.10
**4. Physically, you are starting to feel ready to go back to work.**	Prepared for action—Self-evaluative	3.25 ± 1.02
**5. You have been increasing activities at home to build up strength to go back to work.**	Prepared for action—Behavioral	3.56 ± 1.01
**6. You are getting help from others to return to work.**	Prepared for action—Behavioral	3.23 ± 1.07
**7. You are not ready to go back to work.**	Prepared for action—Self-evaluative	2.81 ± 0.98
**8. You found strategies to make work manageable so you can return to work.**	Prepared for action—Self-evaluative	3.26 ± 0.86
**9. You have been wondering if there is something you could do to return to work.**	Contemplation	3.43 ± 0.98
**10. You have a date for your first day back at work.**	Prepared for action—Self-evaluative	3.15 ± 0.96
**11. You wish you had more ideas about how to get back to work.**	Contemplation	3.58 ± 0.93
**12. You would like to have some advice about how go back to work.**	Contemplation	3.57 ± 0.97
**13. As far as you are concerned, you do not need to go back to work ever.**	Precontemplation	2.61 ± 1.09

RRTW, Readiness to Return-to-Work Scale.

**Table 3:** RRTW scores of PTC survivors who had been back to work (N = 298).

Items	Dimension	Scores (M ± SD)
**14. You are doing everything you can to stay at work.**	Proactive maintenance	4.13 ± 0.88
**15. You learned different ways to cope with your pain so that you can stay at work.**	Proactive maintenance	3.78 ± 1.01
**16. You are taking steps to prevent having to leave work again due to your injury.**	Proactive maintenance	3.81 ± 1.03
**17. You found strategies to make your work manageable so you can stay at work.**	Proactive maintenance	3.96 ± 0.78
**18. You are back at work but not sure you can keep up the effort.**	Uncertain maintenance	3.17 ± 1.13
**19. You worry about having to stop working again due to your injury.**	Uncertain maintenance	3.36 ± 1.15
**20. You still find yourself struggling to stay at work due to the effects of your injury.**	Uncertain maintenance	2.65 ± 1.11
**21. You are back at work, and it is going well.**	Uncertain maintenance	4.04 ± 0.77
**22. You feel you may need help in order to stay at work.**	Uncertain maintenance	2.56 ± 1.14

RRTW, Readiness to Return-to-Work Scale.

Of note, although some non-RTW patients with PTC after surgery, their willingness to work was high. As shown in **Table 4**, nearly 80% of patients were in the prepared-for-action stage, including 91 patients (60.1%) in the prepared for action—self-evaluative (mean score, 3.34 ± 0.56) and 29 patients (19.2%) in the prepared for action—behavioral (mean score, 4.25 ± 0.51). Only 13 patients (8.6%) were in the precontemplation (mean score, 4.08 ± 0.60), and 18 patients (11.9%) were in the contemplation (mean score, 4.31 ± 0.29). Additionally, the proportion of subjects who returned to work in proactive maintenance and uncertain maintenance was similar: 146 (49.0%) *vs*. 152 (51.0%), with mean scores of 3.59 ± 0.57 and 4.19 ± 0.61, respectively (**Table 4**).

**Table 4:** Dimension of RRTW in PTC survivors who were back (N = 298) or not back (N = 151) at work.

RRTW dimension	n (%)	Rank	Score range	Mean of dimension	Mean of items^a^
PTC survivors who were back at work (N = 298)					
Proactive maintenance	146 (49.0)	2	8-25	17.95 ± 2.84	3.59 ± 0.57
Uncertain maintenance	152 (51.0)	1	11-20	16.75 ± 2.45	4.19 ± 0.61
PTC survivors who were not back at work (N = 151)					
Precontemplation	13 (8.6)	4	10-15	12.23 ± 1.79	4.08 ± 0.60
Contemplation	18 (11.9)	3	12-15	12.94 ± 0.87	4.31 ± 0.29
Prepared for action—Self- evaluative	91 (60.1)	1	5-15	13.36 ± 2.25	3.34 ± 0.56
Prepared for action—Behavioral	29 (19.2)	2	9-15	12.76 ± 1.53	4.25 ± 0.51

a Mean score of items contained in each dimension.

Next, we assessed the factors related to RTW in the study population. Our findings revealed that age, gender, education level, alcohol consumption, diet, place of residence, health insurance, postoperative time, type of occupation, monthly income, and postoperative primary caregiver all influenced the decision to RTW among patients with PTC. Specifically, being female, residing in rural areas, lacking health insurance, having a shorter time after surgery, and having a lower income were associated with a higher likelihood of not returning to work (**Supplementary Table S3**).

### 3.4 Detailed assessment of HRQoL status of the study population

To obtain more accurate data on the postoperative HRQoL of patients with PTC, two questionnaires were used together in this study, the EORTC QLQ-C30 and THYCA-QoL. According to the EORTC QLQ-C30, the functioning scale indicated that among the 512 subjects, social function scored the highest, while emotional function scored the lowest. This suggests that emotional function might have a negative impact on QoL. On the symptom scale, fatigue scored the highest, whereas nausea and vomiting scored the lowest. This indicates that patients were more frequently bothered by fatigue compared to other symptoms, potentially leading to a worse QoL. Among the individual items, insomnia scored the highest, suggesting that sleep disturbance had a significant effect on patients (**Table 5**).

**Table 5:** EORTC QLQ-C30 scores of PTC survivors (N = 512).

Scale	Scores (M ± SD)	Rank
Global quality of life^a^	67.19 ± 22.77	
Functioning scale^a^		
Role	82.58 ± 18.97	2
Physical	82.34 ± 13.22	3
Social	83.53 ± 19.78	1
Cognitive	76.07 ± 19.78	4
Emotional	69.29 ± 19.28	5
Symptom scale(item)^b^		
Fatigue	29.49 ± 19.65	1
Pain	15.23 ± 16.06	2
Nausea/vomiting	8.04 ± 13.55	3
Dyspnea	19.47 ± 20.45	4
Loss of appetite	13.28 ± 19.49	5
Insomnia	24.09 ± 27.53	1
Constipation	23.18 ± 24.34	2
Diarrhea	11.26 ± 18.57	6
Financial difficulties	19.79 ± 25.00	3

a A higher score indicates better functioning.

b A higher score indicates more symptoms.

EORTC QLQ-C30, European Organization for Research and Treatment of Cancer Quality of Life Questionnaire.

Furthermore, the THYCA-QoL scale revealed that in the symptom scales, voice scored the highest, while concentration scored the lowest. Among the single items, chills and scars ranked first and second, respectively, while tingling in the arms and legs scored the lowest. Overall, these data indicate that patients with PTC were significantly affected by their voice condition, chills, and scars (**Table 6**).

**Table 6:** THYCA-QoL scores of PTC survivors (N = 512).

Scale	Scores (M ± SD)	Rank
Symptom scale^a^		
Neuromuscular	20.40 ± 16.60	4
Voice	31.87 ± 28.83	1
Concentration	16.44 ± 19.88	7
Sympathetic	20.38 ± 19.23	5
Throat/mouth	23.29 ± 17.28	3
Psychological	24.67 ± 18.65	2
Sensory	20.25 ± 17.76	6
Single items^a^		
Scar	22.20 ± 29.27	2
Chilly	25.52 ± 25.45	1
Tingling	11.65 ± 19.25	6
Weight gain	19.08 ± 24.20	4
Headache	17.84 ± 21.03	5
Sex^b^	20.70 ± 22.19	3

a A higher score indicates more symptoms.

b A higher score indicates better functioning.

THYCA–QOL, Thyroid Cancer–Specific Quality of Life Questionnaire.

Considering the close relationship between psychological state and QoL, we further explored the impact of anxiety on HRQoL state in PTC patients after surgery. Firstly, according to EORTC QLQ-C30, as we expected, all of the anxious patients had poorer QoL than non-anxious patients in each of the five functional scales, three symptom scales, and six single items (**Table 7**, **Supplementary Figure S2A**). In terms of the functional scale, both anxious and non-anxious patients had the lowest scores for emotional functioning (81.22 ± 13.29 and 57.98 ± 17.11), indicating poor emotional well-being in both groups. Patients without anxiety had the highest score for social functioning (91.97 ± 14.57), while patients with anxiety had the highest score for physical functioning (78.43 ± 14.24). Additionally, regarding the symptom scale, fatigue was the most prominent symptom since it showed the highest score in both anxious and non-anxious patients (20.53 ± 15.65 and 37.98 ± 19.23, respectively). Whereas, according to the six single items, the anxious and non-anxious groups experienced differently; the anxious patients were bothered by insomnia most (scored 34.22 ± 29.55), and the non-anxious group was bothered by constipation most (scored 18.21 ± 21.72). Then, according to THYCA QoL, all anxious patients had lower HRQoL than those without anxiety in both seven-symptom-scale and six-single-item. On the symptom scale, both patients with and without anxiety scored the highest in voice (25.64 ± 27.46 and 37.77 ± 28.80, **Table 8**, **Supplementary Figure S2B**); while among the six single items, the non-anxious group had the highest score in sexual interest.

**Table 7:** EORTC QLQ-C30 scores of all dimensions in patients with and without anxiety (N = 512).

Scale	Nonanxiety		Anxiety	
	Scores (M ± SD)	Rank	Scores (M ± SD)	Rank
Global quality of life^a^	75.80 ± 20.56		59.03 ± 21.68	
Functioning scale^a^				
Role	89.36 ± 15.07	2	76.17 ± 19.99	2
Physical	86.48 ± 10.54	3	78.43 ± 14.24	1
Social	91.97 ± 14.57	1	75.54 ± 20.69	3
Cognitive	85.68 ± 13.69	4	66.98 ± 20.31	4
Emotional	81.22 ± 13.29	5	57.98 ± 17.11	5
Symptom scale(item)^b^				
Fatigue	20.53 ± 15.65	1	37.98 ± 19.23	1
Pain	9.37 ± 11.63	2	20.78 ± 17.59	2
Nausea/vomiting	4.02 ± 8.81	3	11.85 ± 15.92	3
Dyspnea	13.79 ± 18.46	2	24.84 ± 20.75	4
Loss of appetite	8.30 ± 15.31	5	18.00 ± 21.69	5
Insomnia	13.39 ± 20.24	3	34.22 ± 29.55	1
Constipation	18.21 ± 21.72	1	27.88 ± 25.67	2
Diarrhea	6.96 ± 14.50	6	15.34 ± 20.89	6
Financial difficulties	12.45 ± 20.94	4	26.74 ± 26.46	3

a A higher score indicates better functioning.

b A higher score indicates more symptoms.

EORTC QLQ-C30, European Organization for Research and Treatment of Cancer Quality of Life Questionnaire.

**Table 8:** THYCA-QoL scores of all dimensions in patients with and without anxiety (N = 512).

Scale	Nonanxiety		Anxiety	
	Scores (M ± SD)	Rank	Scores (M ± SD)	Rank
Symptom scale^a^				
Neuromuscular	13.79 ± 12.70	5	26.66 ± 17.37	4
Voice	25.64 ± 27.46	1	37.77 ± 28.80	1
Concentration	7.30 ± 12.47	7	25.10 ± 21.60	7
Sympathetic	14.46 ± 15.03	3	25.98 ± 20.98	5
Throat/mouth	18.34 ± 15.34	2	27.97 ± 17.68	3
Psychological	13.02 ± 11.30	6	35.71 ± 17.45	2
Sensory	14.46 ± 14.43	4	25.73 ± 18.82	6
Single items^a^				
Scar	13.65 ± 24.11	4	30.29 ± 31.29	2
Chilly	17.27 ± 20.93	3	33.33 ± 26.80	1
Tingling	6.29 ± 14.35	6	16.73 ± 21.72	6
Weight gain	16.33 ± 22.58	2	21.67 ± 25.32	4
Headache	11.38 ± 16.36	5	23.95 ± 23.00	3
Sex^b^	22.62 ± 22.78	1	18.89 ± 21.42	5

a A higher score indicates more symptoms.

b A higher score indicates better functioning.

THYCA–QOL, Thyroid Cancer–Specific Quality of Life Questionnaire.

Furthermore, we also investigated the influence of RTW status on HRQoL in PTC patients after surgery (**Tables 9** and **10**, **Supplementary Figures S2C, D**). On the functional scale of the EORTC QLQ-C30, the non-RTW patients had the highest score on physical functioning (79.51 ± 14.21; **Table 9**), while the RTW patients had the highest score on social functioning (86.13 ± 17.37). The lowest score in both groups was emotional functioning (67.00 ± 21.16 and 69.13 ± 18.04). On the symptom scale, fatigue scored highest in both groups (32.30 ± 18.90 and 27.37 ± 19.59); nausea/vomiting had the lowest scores (10.38 ± 15.43 and 6.99 ± 12.76). In the non-RTW group, the highest score was in financial difficulties (27.15 ± 29.32); while in the RTW group, the highest score was in insomnia (21.92 ± 26.83). In addition, the results of THYCA-QoL showed all RTW patients experienced lower QoL than the non-RTW patients according to all seven symptom scales (**Table 10**), especially on voice-related symptoms (both groups scored the highest on voice (39.96 ± 31.76 in non-RTW group and 26.85 ± 26.56 in RTW group)). Among the six single items, the non-RTW group had the highest score for feeling chilly (27.81 ± 26.14), while the RTW group had the highest score in sexual interest (25.28 ± 22.40).

**Table 9:** EORTC QLQ-C30 scores of all dimensions in patients whether they returned to work or not (N = 449).

Scale	Not returned to work		Returned to work	
	Scores (M ± SD)	Rank	Scores (M ± SD)	Rank
Global quality of life^a^	59.49 ± 26.58		70.64 ± 18.54	
Functioning scale^a^				
Role	76.71 ± 20.49	3	85.40 ± 17.44	2
Physical	79.51 ± 14.21	1	84.09 ± 12.51	3
Social	78.81 ± 23.25	2	86.13 ± 17.37	1
Cognitive	75.83 ± 20.36	4	75.67 ± 19.64	4
Emotional	67.00 ± 21.16	5	69.13 ± 18.04	5
Symptom scale (item)^b^				
Fatigue	32.30 ± 18.90	1	27.37 ± 19.59	1
Pain	20.31 ± 17.78	2	12.14 ± 14.35	2
Nausea/vomiting	10.38 ± 15.43	3	6.99 ± 12.76	3
Dyspnea	22.74 ± 20.80	4	17.56 ± 20.46	3
Loss of appetite	15.01 ± 19.06	5	11.30 ± 18.19	6
Insomnia	25.83 ± 28.24	2	21.92 ± 26.83	1
Constipation	25.61 ± 25.57	3	20.69 ± 22.86	2
Diarrhea	10.82 ± 17.81	6	11.86 ± 19.14	5
Financial difficulties	27.15 ± 29.32	1	16.11 ± 21.54	4

a A higher score indicates better functioning.

b A higher score indicates more symptoms.

EORTC QLQ-C30, European Organization for Research and Treatment of Cancer Quality of Life Questionnaire.

**Table 10:** THYCA-QoL scores of all dimensions in patients whether back at work or not (N = 449).

Scale	Not returned to work		Returned to work	
	Scores (M ± SD)	Rank	Scores (M ± SD)	Rank
Symptom scale^a^				
Neuromuscular	22.44 ± 17.13	5	18.87 ± 16.70	5
Voice	39.96 ± 31.76	1	26.85 ± 26.56	1
Concentration	17.66 ± 22.31	7	15.83 ± 18.63	7
Sympathetic	22.52 ± 20.32	4	18.23 ± 18.10	6
Throat/mouth	28.77 ± 19.28	2	20.32 ± 15.87	3
Psychological	27.21 ± 20.04	3	23.83 ± 17.67	2
Sensory	20.20 ± 18.16	6	20.25 ± 18.22	4
Single items^a^				
Scar	25.61 ± 31.50	2	21.03 ± 29.12	3
Chilly	27.81 ± 26.14	1	24.61 ± 23.62	2
Tingling	11.48 ± 18.01	6	11.41 ± 19.98	6
Weight gain	19.21 ± 25.00	4	20.58 ± 24.76	4
Headache	20.98 ± 23.21	3	15.32 ± 20.07	5
Sex^b^	17.66 ± 21.64	5	25.28 ± 22.40	1

a A higher score indicates more symptoms.

b A higher score indicates better functioning.

THYCA–QOL, Thyroid Cancer–Specific Quality of Life Questionnaire.

### 3.5 Multivariate logistic regression model to predict anxiety occurrence in PTC patients after surgery

**Figure 2:**
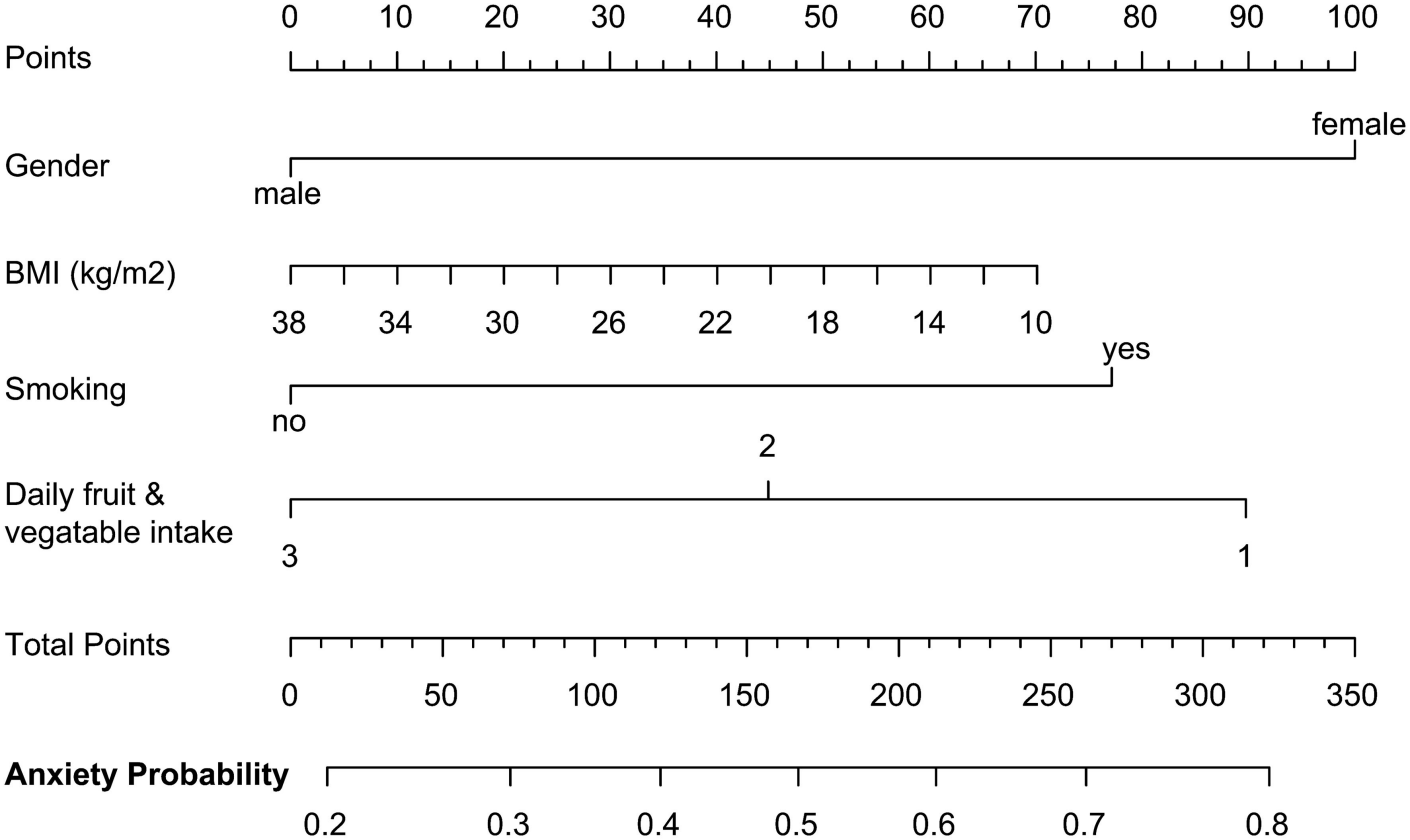
Nomogram to predict anxiety occurrence in PTC patients after surgery. The nomogram to predict anxiety-risk is based on the above four predictive factors. To use the nomogram, the value of each factor is placed on each variable axis and a line is drawn upward to determine the number of received points for each value of the factor. The sum of these values is located on the total point axis and a line down the bottom axis is drawn to determine the probability of anxiety.

To assist in the clinical diagnosis and intervention of anxiety in advance, we established a multivariate logistic regression model to predict the occurrence of anxiety in PTC patients after surgery. Our data revealed that gender was an independent risk factor for anxiety, with female patients being more likely to experience anxiety symptoms than males [*P* = 0.002, OR (95% CI) = 2.396 (1.389-4.135), **Table 11**]. Additionally, some indicators such as nonsmoking, higher daily intake of fruits and vegetables, and higher BMI were also identified as potential risk factors for anxiety occurrence (**Table 11**). The anxiety-risk estimation nomogram was developed using these independent predictors, as depicted in **Figure 2**. The nomogram is based on the coefficients from our logistic regression analysis, providing a user-friendly method for clinicians to estimate a risk for anxiety for patients.

**Table 11:** Multivariate logistic regression model to predict anxiety in PTC patients after surgery (N = 512).

Variables	β	S.E.	Wald	p-Value	OR (95% CI)
**Gender (female *vs.* male)**	0.874	0.278	9.857	0.002	2.396 (1.389-4.135)
**Smoking (no *vs.* yes)**	-0.696	0.375	3.448	0.063	0.499 (0.239-1.039)
**Daily fruit and vegetable intake (per 1 serving increase)**	-0.406	0.168	5.850	0.016	0.667 (0.480-0.926)
**BMI (per 0.01 kg/m^2^ increase)**	-0.034	0.027	1.669	0.196	0.966 (0.917-1.018)
**Constant**	1.253	0.725	1.731	0.045	4.698

### 3.6 Multivariate logistic regression model to predict RRTW in PTC patients after surgery

**Figure 3:**
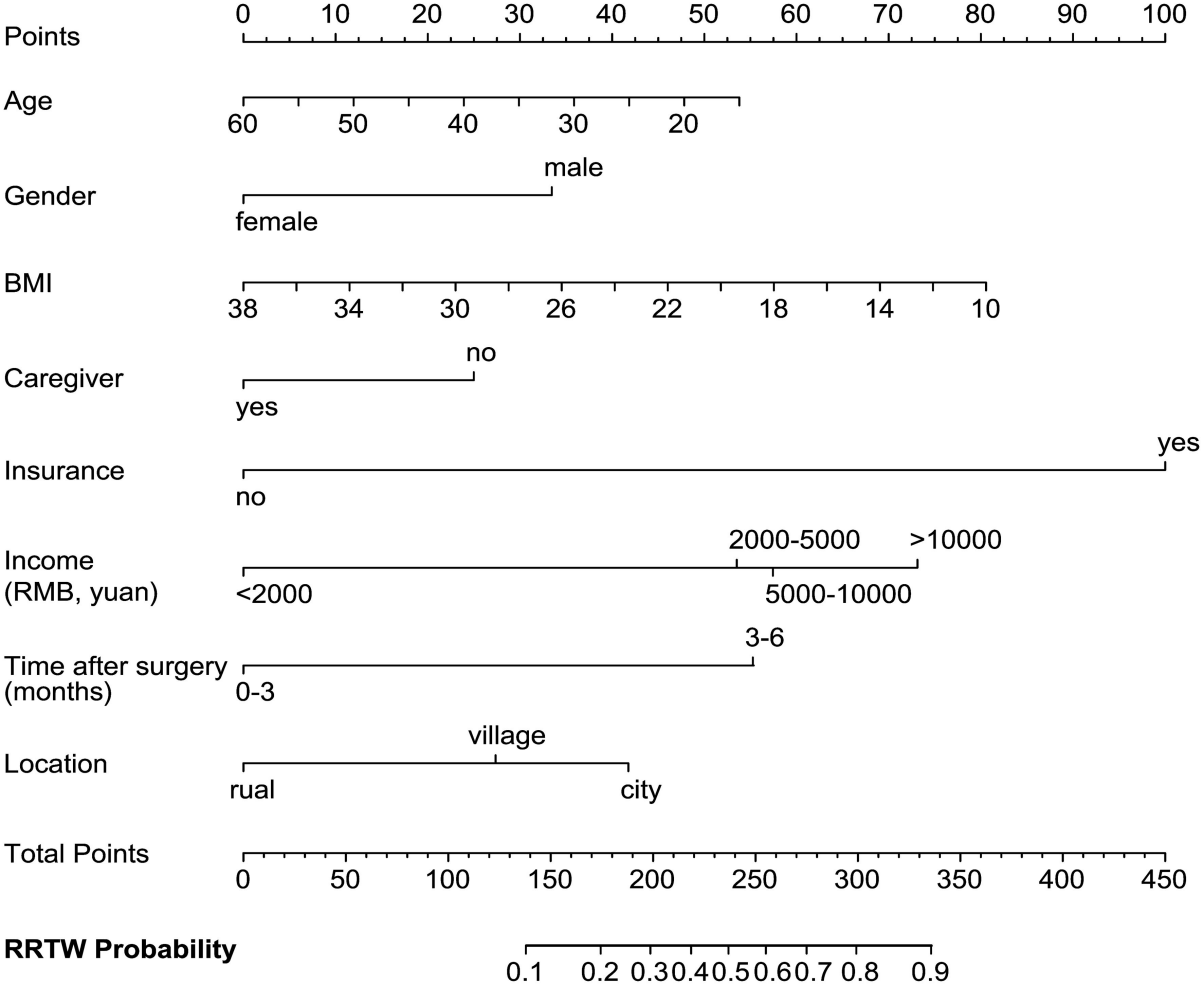
Nomogram to predict RRTW in PTC patients after surgery. Each predictive variable, such as age, gender, BMI, caregiver, health insurance, monthly income, time after surgery, and residence, is assigned a point value based on its coefficient in the logistic regression model. To use the nomogram, the value of each factor is placed on each variable axis and a line is drawn upward to determine the number of received points for each value of the factor. The total points accrued from all predictive variables are used to determine the probability of RTW, as indicated on the scale at the bottom of the nomogram.

Similarly, to facilitate the early return to normal work for patients, we conducted a multivariate logistic regression model to predict the level of readiness. The data revealed that being female, having a higher BMI, lacking health insurance, and older age were independent risk factors for not returning to work. On the other hand, having a caregiver, higher monthly income, longer time after surgery, and residing in urban areas were positive factors for early RTW (**Table 12**). These independent predictors were used to form the RRTW estimation nomogram, as shown in **Figure 3**. To use the nomogram in a clinical setting, the values of each factor needs to be added and the total score of all predictors would be applied to determine the probability of RRTW.

**Table 12:** Multivariate logistic regression model to predict RRTW in PTC patients after surgery (N = 449).

Variables	β	S.E.	Wald	p-Value	OR (95% CI)
**Age (years, for per 1 year increase)**	-0.321	0.126	6.523	0.011	0.725 (0.567-0.928)
**Gender (female *vs.* male)**	-0.657	0.340	3.737	0.053	0.519 (0.266-1.009)
**BMI (per 0.01 kg/m^2^ increase)**	-0.064	0.031	-2.043	0.042	0.731 (0.541-0.988)
**Caregiver (no *vs*. yes)**	0.531	0.271	3.840	0.050	1.701 (1.000-2.892)
**Health insurance (no *vs*. yes)**	-2.339	0.960	5.933	0.015	0.096 (0.015-0.633)
Monthly income^a^ (yuan)					
**b *vs.* a**	1.242	0.305	16.566	<0.01	3.463 (1.904-6.297)
**c *vs.* a**	1.628	0.387	17.701	<0.01	5.094 (2.386-10.875)
**d *vs.* a**	1.385	0.616	5.048	0.025	3.994 (1.193-13.367)
**Time after surgery (for each 3-month increment)**	1.253	0.250	25.214	<0.01	3.501 (2.147-5.710)
Residence^b^					
**b *vs.* a**	0.907	0.398	5.195	0.023	2.477 (1.135-5.403)
**c *vs.* a**	0.589	0.315	3.499	0.061	1.801 (0.972-3.337)
**Constant**	1.727	0.973	1.771	0.076	

a different categories of monthly income; a, <2000; b, 2000-5000; c, 5000-10000; d, >10000.

b different categories of residence area; a, rural area; b, villages; c, city.

## 4 Discussion

The incidence of PTC in young adults has increased over the years (45), and the 10-year survival rate of PTC patients after surgical treatment is over 90% (46). There is a significant need to enhance HRQoL in this population (47). In the present study, a total of 512 young PTC patients who underwent surgery at our center were included retrospectively; and based on the assessment of physical and psychological symptoms, the ability to return to work was evaluated systematically. Our study comprehensively assessed the postoperative QoL of PTC patients and related influencing factors across multiple dimensions, including post-thyroid cancer-specific symptoms, general post-cancer symptoms, psychological anxiety status, and readiness to return to work, using four follow-up questionnaires and a ‘step-by-step’ evaluation strategy.

As reported in recent studies, voice problems and scarring were the most prominent specific symptoms associated with thyroid surgery (26, 48). Whether using traditional open surgery or minimally invasive thyroidectomy, it’s difficult to completely avoid the disruption to the recurrent laryngeal nerve (49–51), which could lead to transient voice changes with various degrees in postoperative patients. Therefore, surgeons still need to care for the protection of the recurrent laryngeal nerve and promote rapid recovery of the patient’s voice after surgery. Additionally, scarring is an inevitable issue for almost all PTC patients, especially for female patients who have a greater concern for neck scarring (52, 53). Despite there are various surgical methods available, traditional open surgery remains one of the primary surgical approaches. In recent years, an increasing number of patients prefer to undergo minimally invasive thyroidectomy, including endoscopic and robotic thyroidectomy (54). However, it’s important to note that post-operative QoL for PTC patients undergoing minimally invasive thyroidectomy has not been shown to improve significantly compared to those undergoing traditional open surgery (55). Postoperative adhesions caused by endoscopic and robotic thyroidectomy, including internal and external scarring, could still lead to upper gastrointestinal and respiratory symptoms, affecting patients’ QoL (56). Whether traditional open or minimally invasive surgery was given, the prevention of scarring and adhesions would still be an area of concern at the current stage.

It is reported that social functioning, fatigue, and insomnia are important factors affecting postoperative HRQoL among patients who have undergone cancer operations (24, 57, 58). Similarly, in this study, PTC patients also exhibited the above three symptoms. Besides that, insomnia is prevalent among PTC patients, which may be related to psychological factors; as a considerable portion of this group experience psychological disorders such as anxiety. More importantly, it cannot be excluded that suppressive levothyroxine therapy may exert an impact on postoperative HRQoL (59). Subclinical hyperthyroidism, characterized by suppressed thyroid-stimulating hormone (TSH) levels, may directly affect the sleep quality of patients with PTC (60). Additionally, the accompanying side effects, such as increased heart rate, may also contribute to sleep disturbances in patients (61). Therefore, it is suggested that precise and individualized TSH suppression treatments are important in TSH suppression therapy to improve postoperative symptoms in PTC patients.

Furthermore, our study indicated that the PTC patients experienced significant psychological anxiety while experiencing subjective symptoms and physical discomforts. In our studied population, more than 50% of PTC patients experienced varying degrees of anxiety, which may be related to the significant psychological burden of the disease, partly due to misconceptions about PTC (62). Furthermore, our research indicates that anxiety is more likely to occur in female patients, possibly due to their higher psychological sensitivity and susceptibility to social functions, vocational issues, and lifestyle factors (16, 28, 63, 64). Therefore, more clinical attention is deserved in postoperative psychological assessment and management for female patients with PTC. Collectively, we recommend that focusing on mental health as a key aspect of postoperative management is crucial to prevent the impact of anxiety on patients’ work and daily life, especially for female patients (28). The study by Chen et al. mentioned that sexual interest was impaired in patients after PTC (65). Interestingly, there was no significant impact on sexual interest reported in our study population, and it could be due to the fact that our study population was mainly composed of young and middle-aged adults, who remained more sexually active.

In current social life, protecting the workability of young and middle-aged PTC patients is of great significance, especially given the widespread potential overdiagnosis and overtreatment of PTC (45, 66). In our study, the majority of PTC patients expressed a strong willingness to return to work after undergoing surgical treatment. However, it was important to note that among those who returned to work, the number of individuals in PM and UM was similar. This may be related to the symptoms such as fatigue experienced by postoperative patients. Despite the low invasive nature of PTC, these symptoms further exacerbated their concerns about the disease itself, thereby inducing anxiety about their current occupational responsibilities (67). In contrast, compared to those who did not return to work, those who did reported better QoL in terms of social interactions, family life, and economic burden. Therefore, appropriate encouragement to guide them back to work is beneficial for PTC patients, which would help them gain more social support and satisfaction, thereby improving their QoL. Specifically, regarding young and middle-aged patients, the vocational capacity of this group holds significant practical value in contemporary society, necessitating further attention to the impact of reduced quality of life resulting from PTC on their reintegration into the workforce.

Previous studies primarily focus on evaluating either postoperative QoL (26–29) or assessing the postoperative psychological status of patients (30–33), while neglecting other important dimensions at the same time. Therefore, a comprehensive study containing analysis across multiple dimensions and their related factors in PTC patients after surgery is still necessary. In this study, we have conducted detailed investigations from three aspects: psychological state, RRTW, and QoL after surgery using four different scales simultaneously. To provide a more comprehensive evaluation of the patient’s QoL after surgery, we employed two scales concurrently: EORTC QLQ-C30 and THYCA-QoL. The former is widely used for assessing the basic QoL in the general population, while the latter is specifically designed for TC patients. By combining these two scales, we gained a better understanding of the study population’s QoL and facilitated the development of a more rational postoperative management strategy for PTC. Additionally, to guide timely postoperative intervention and enhance clinical capabilities in managing PTC patients after surgery, we investigated factors influencing postoperative psychological anxiety and return to work. Various statistical methods were appropriately applied in this analysis; initially conducting univariate analysis on influencing factors followed by multivariate logistic regression analysis to evaluate the simultaneous impact of multiple variables on outcomes. More importantly, to visualize our study results, we conducted a nomogram for clinical application, which could help clinicians develop personalized and reasonable postoperative management measures. In summary, our study provides valuable insights that were lacking in previous similar studies (16, 17, 24, 27, 65), and contributes to the enhancement of clinical guidelines for promoting comprehensive recovery among post-thyroidectomy patients.

Despite the intriguing findings of this study, several limitations should be acknowledged. First, the study only included patients with PTC, and it is necessary to investigate patients with other types of TC in future research. Second, the follow-up duration in this study was relatively short, and a longer-term follow-up is needed to assess the long-term outcomes. Third, this is a cross-sectional study, causal relationships between the outcome variables and HRQoL cannot be inferred. Thus, a prospective study design would be required in the following research. Indeed, we are currently conducting a longitudinal study in a multicenter with more types of TC samples and a more extended follow-up period, expecting to provide more personalized therapeutic intervention strategies for the postoperative rehabilitation of a larger population of TC patients in the future.

## 5 Conclusion

In conclusion, this study has systemically accessed the psychological status, HRQoL, and RTW state, as well as their associated factors in more than 500 young and middle-aged PTC patients. The findings of our study demonstrate that early and comprehensive assessments, combined with the development of personalized and efficacious intervention and treatment strategies, would contribute to enhancing their HRQoL and restoring their vocational capacity. This study has the potential to provide valuable insights for timely interventions in postoperative PTC patients and offer clinical guidance to promote their recovery from surgery.

## Data availability statement

The raw data supporting the conclusions of this article will be made available by the authors, without undue reservation.

## Ethics statement

The studies involving humans were approved by Medical Ethics Committee of the Second affiliated hospital, Air Force Medical University. The studies were conducted in accordance with the local legislation and institutional requirements. The participants provided their written informed consent to participate in this study. Written informed consent was obtained from the individual(s) for the publication of any potentially identifiable images or data included in this article.

## Author contributions

SC: Data curation, Writing – original draft, Writing – review & editing. XH: Investigation, Writing – review & editing. PY: Investigation, Writing – review & editing. LY: Data curation, Methodology, Writing – review & editing. SP: Formal Analysis, Methodology, Writing – review & editing. LH: Data curation, Writing – review & editing. LJY: Data curation, Writing – review & editing. GB: Funding acquisition, Project administration, Writing – review & editing.
