# Supplementary material for: Status of mental and social activities of young and middle-aged patients after papillary thyroid cancer surgery
Source: Front Oncol. 2024 Mar 26;14:1338216. doi: 10.3389/fonc.2024.1338216 (PMC11002165; doi:10.3389/fonc.2024.1338216)
Supplement: Supplementary file 1 [file DataSheet_1.docx]

Supplementary Material

# Supplementary Data

# Supplementary Tables

**Table S1.** Basic characteristics of participants with PTC (N=512)

| Characteristics | Grouping | n | % |
| --- | --- | --- | --- |
| Age (years) | 19-30 | 78 | 15.2 |
|  | 31-40 | 156 | 30.5 |
|  | 41-50 | 148 | 28.9 |
|  | 51-60 | 130 | 25.4 |
| Gender | male | 96 | 18.8 |
|  | female | 416 | 81.3 |
| BMI (kg/m^2^) | <18.5 | 38 | 7.4 |
|  | 18.5-23.9 | 272 | 53.1 |
|  | 24-27.9 | 163 | 31.8 |
|  | ≥28 | 39 | 7.6 |
| Ethnicity | Han | 506 | 98.8 |
|  | other | 6 | 1.2 |
| Education | primary school and less | 21 | 4.1 |
|  | junior high school | 100 | 19.5 |
|  | high school | 98 | 19.1 |
|  | junior college | 118 | 23.0 |
|  | Bachelor’s degree or more | 175 | 34.2 |
| Smoking | yes | 46 | 9.0 |
|  | no | 466 | 91.0 |
| Drinking | yes | 69 | 13.5 |
|  | no | 443 | 86.5 |
| Dietary status | light taste | 116 | 22.7 |
|  | over taste | 68 | 13.3 |
|  | normal | 328 | 64.1 |
| Residence | rural area | 90 | 17.6 |
|  | villages | 70 | 13.7 |
|  | city | 352 | 68.8 |
| Marital status | unmarried | 35 | 6.8 |
|  | married | 461 | 90.0 |
|  | divorced | 9 | 1.8 |
|  | widowed | 7 | 1.4 |
| Health insurance | yes | 502 | 98.0 |
|  | no | 10 | 2.0 |
| Months after surgery | 1-3 | 269 | 52.5 |
|  | 3-6 | 243 | 47.5 |
| Occupation | employed or student | 292 | 57.0 |
|  | unemployed | 174 | 34.0 |
|  | retired | 46 | 9.0 |
| Monthly income | <2000 | 136 | 26.6 |
|  | 2000-5000 | 236 | 46.1 |
|  | 5000-10000 | 114 | 22.3 |
|  | >10000 | 26 | 5.1 |
| Caregiver | yes | 358 | 69.9 |
|  | no | 154 | 30.1 |
| Operation | open | 330 | 64.5 |
|  | endoscope | 182 | 35.5 |
| Scope of surgery | unilateral | 171 | 33.4 |
|  | bilateral | 81 | 15.8 |
|  | thyroidectomy and lymphatic dissection | 260 | 50.8 |
| Levothyroxine | yes | 504 | 98.4 |
|  | no | 8 | 1.6 |
| Moderate physical activity per week | 0 | 74 | 14.5 |
|  | 1-2 | 196 | 38.3 |
|  | 3-4 | 128 | 25.0 |
|  | ≥5 | 114 | 22.3 |
| Daily fruit and vegetable intake (portions^#^) | <1 | 94 | 18.4 |
|  | 1-2 | 358 | 69.9 |
|  | 3-4 | 60 | 11.7 |

PTC, papillary thyroid carcinoma; BMI, body mass index.

^#^ Fruit and vegetable intake was evaluated with a separate item asking for the number of servings of fruit and vegetable consumed per day (including examples of servings).

**Table S2.** The factors influencing anxiety in the study population (N = 512)

| Characteristics | Grouping | Anxiety | | χ^2^ | *P* |
| --- | --- | --- | --- | --- | --- |
|  |  | No  n = 249 (48.6%) | Yes  n = 263 (51.4%) |  |  |
| Age (years) | 19-30 | 41 (16.5) | 37 (14.1) | 8.191 | **0.042**^*^ |
|  | 31-40 | 61 (24.5) | 95 (36.1) |  |  |
|  | 41-50 | 79 (31.7) | 69 (26.2) |  |  |
|  | 51-60 | 68 (27.3) | 62 (23.6) |  |  |
| Gender | male | 59 (23.7) | 37 (14.1) | 7.780 | **< 0.01^**^** |
|  | female | 190 (76.3) | 226 (85.9) |  |  |
| BMI (kg/m^2^) | <18.5 | 12 (4.8) | 26 (9.9) | 7.62 | 0.055 |
|  | 18.5-23.9 | 135 (54.2) | 137 (52.1) |  |  |
|  | 24-27.9 | 87 (34.9) | 76 (28.9) |  |  |
|  | ≥28 | 15 (6.0) | 24 (9.1) |  |  |
| Ethnicity | Han | 248 (99.6) | 258 (98.1) | 1.357 | 0.244 |
|  | other | 1 (0.4) | 5 (1.9) |  |  |
| Education | primary school and less | 9 (3.6) | 12 (4.6) | 0.785 | 0.940 |
|  | junior high school | 48 (19.3) | 52 (19.8) |  |  |
|  | high school | 47 (18.9) | 51 (19.4) |  |  |
|  | junior college | 61 (24.5) | 57 (21.7) |  |  |
|  | Bachelor’s degree or more | 84 (33.7) | 91 (34.6) |  |  |
| Smoking | yes | 21 (8.4) | 25 (9.5) | 0.180 | 0.672 |
|  | no | 228 (91.6) | 238 (90.5) |  |  |
| Drinking | yes | 31 (12.4) | 38 (14.4) | 0.438 | 0.508 |
|  | no | 218 (87.6) | 225 (85.6) |  |  |
| Dietary status | light taste | 53 (21.3) | 63 (24.0) | 2.794 | 0.247 |
|  | over taste | 28 (11.2) | 40 (15.2) |  |  |
|  | normal | 168 (67.5) | 160 (60.8) |  |  |
| Residence | rural area | 43 (17.3) | 47 (17.9) | 0.126 | 0.939 |
|  | villages | 33 (13.3) | 37 (14.1) |  |  |
|  | city | 173 (69.5) | 179 (68.1) |  |  |
| Marital status | unmarried | 18 (7.2) | 17 (6.5) | 1.671 | 0.643 |
|  | married | 222 (89.2) | 239 (90.9) |  |  |
|  | divorced | 4 (1.6) | 5 (1.9) |  |  |
|  | widowed | 5 (2.0) | 2 (0.8) |  |  |
| Health insurance | yes | 247 (99.2) | 255 (97.0) | 2.280 | 0.131 |
|  | no | 2 (0.8) | 8 (3.0) |  |  |
| Months after surgery | 1-3 | 133 (53.4) | 136 (51.7) | 0.149 | 0.700 |
|  | 3-6 | 116 (46.6) | 127 (48.3) |  |  |
| Occupation | employed or student | 158 (63.5) | 152 (57.8) | 1.899 | 0.387 |
|  | unemployed | 69 (27.7) | 87 (33.1) |  |  |
|  | retired | 22 (8.8) | 24 (9.1) |  |  |
| Monthly income | <2000 | 64(25.7) | 72(27.4) | 1.675 | 0.642 |
|  | 2000-5000 | 111(44.6) | 125(47.5) |  |  |
|  | 5000-10000 | 59(23.7) | 55(20.9) |  |  |
|  | >10000 | 15(6.0) | 11(4.2) |  |  |
| Caregiver | yes | 177 (71.1) | 181 (68.8) | 0.311 | 0.577 |
|  | no | 72 (28.9) | 82 (31.2) |  |  |
| Operation | open | 165 (66.3) | 165 (62.7) | 0.695 | 0.405 |
|  | endoscope | 84 (33.7) | 98 (37.3) |  |  |
| Scope of surgery | unilateral | 80 (32.1) | 91 (34.6) | 2.574 | 0.276 |
|  | bilateral | 46 (18.5) | 35 (13.3) |  |  |
|  | thyroidectomy and lymphatic dissection | 123 (49.4) | 137 (52.1) |  |  |
| Levothyroxine | yes | 247 (99.2) | 257 (97.7) | 1.817 | 0.178 |
|  | no | 2 (0.8) | 6 (2.3) |  |  |
| Daily activities/days | 0 | 38 (15.3) | 36 (13.7) | 0.717 | 0.869 |
|  | 1-2 | 93 (37.3) | 103 (39.2) |  |  |
|  | 3-4 | 60 (24.1) | 68 (25.9) |  |  |
|  | ≥5 | 58 (23.3) | 56 (21.3) |  |  |
| Daily fruit and vegetable intake (portions^#^) | <1 | 37 (14.9) | 57 (21.7) | 6.378 | **0.041**^*^ |
|  | 1-2 | 176 (70.7) | 182 (69.2) |  |  |
|  | 3-4 | 36 (14.5) | 24 (9.1) |  |  |

Bold values indicate statistical significance (^*^*P* <0.05; ^**^*P* <0.*01; ^***^P* <0.001).

^#^ Fruit and vegetable intake was evaluated with a separate item asking for the number of servings of fruit and vegetable consumed per day (including examples of servings).

**Table S3.** The factors influencing return to work in the study population (N = 449)

| Characteristics | Grouping | Return to work | | χ^2^ | *P* |
| --- | --- | --- | --- | --- | --- |
|  |  | Yes  n = 298 (66.4%) | No  n = 151 (33.6%) |  |  |
| Age (years) | 19-30 | 53 (17.8) | 25 (16.6) | 15.053 | **< 0.01**^**^ |
|  | 31-40 | 118 (39.6) | 38 (25.2) |  |  |
|  | 41-50 | 92 (30.9) | 53 (35.1) |  |  |
|  | 51-60 | 35 (11.7) | 35 (23.2) |  |  |
| Gender | male | 77 (25.8) | 17 (11.3) | 12.872 | **﹤0.001**^***^ |
|  | female | 190 (76.3) | 226 (85.9) |  |  |
| BMI (kg/m^2^) | <18.5 | 28 (9.4) | 5 (3.3%) | 11.970 | **< 0.01**^**^ |
|  | 18.5-23.9 | 154 (51.7) | 84 (55.6) |  |  |
|  | 24-27.9 | 99 (33.2) | 43 (28.5) |  |  |
|  | ≥28 | 17 (5.7) | 19 (12.6) |  |  |
| Ethnicity | Han | 293 (98.3) | 150 (99.3) | 0.784 | 0.376 |
|  | other | 5 (1.7) | 1 (0.7) |  |  |
| Education | primary school and less | 5 (1.7) | 13 (8.6) | 41.841 | **﹤0.001^***^** |
|  | junior high school | 38 (12.8) | 42 (27.8) |  |  |
|  | high school | 40 (13.4) | 30 (19.9) |  |  |
|  | junior college | 78 (26.2) | 30 (19.9) |  |  |
|  | Bachelor’s degree or more | 137 (46.0) | 36 (23.8) |  |  |
| Smoking | yes | 31 (10.4) | 14 (9.3) | 0.142 | 0.706 |
|  | no | 267 (89.6) | 137 (90.7) |  |  |
| Drinking | yes | 53 (17.8) | 15 (9.9) | 4.807 | **0.028^*^** |
|  | no | 245 (82.2) | 136 (90.1) |  |  |
| Dietary status | light taste | 49 (16.4) | 42 (27.8) | 8.887 | **0.012^*^** |
|  | over taste | 42 (14.1) | 23 (15.2) |  |  |
|  | normal | 207 (69.5) | 86 (57.0) |  |  |
| Residence | rural area | 37 (12.4) | 50 (33.1) | 27.688 | **﹤0.001^***^** |
|  | villages | 46 (15.4) | 20 (13.2) |  |  |
|  | city | 215 (72.1) | 81 (53.6) |  |  |
| Marital status | unmarried | 24 (8.1) | 11 (7.3) | 0.367 | 0.947 |
|  | married | 266 (89.3) | 137 (90.7) |  |  |
|  | divorced | 6 (2.0) | 2 (1.3) |  |  |
|  | widowed | 2 (0.7) | 1 (0.7) |  |  |
| Health insurance^a^ | yes | 296 (99.3) | 144 (95.4) | 6.128 | **0.013^*^** |
|  | no | 2 (0.7) | 7 (4.6) |  |  |
| Time after surgery (months) | 1-3 | 132 (44.3) | 106 (70.2) | 26.996 | **﹤0.001^**^****^*^** |
|  | 3-6 | 166 (55.7) | 45 (29.8) |  |  |
| Occupation | employed or student | 230 (77.2) | 80 (53.0) |  |  |
|  | unemployed | 68 (22.8) | 71 (47.0) |  |  |
| Monthly income | <2000 | 43 (14.4) | 64 (42.4) | 46.215 | **﹤0.001^**^****^*^** |
|  | 2000-5000 | 148 (49.7) | 60 (39.7) |  |  |
|  | 5000-10000 | 89 (29.9) | 21 (13.9) |  |  |
|  | >10000 | 18 (6.0) | 6 (4.0) |  |  |
| Caregiver | yes | 196 (65.8) | 117 (77.5) | 6.510 | **0.011^*^** |
|  | no | 102 (34.2) | 34 (22.5) |  |  |
| Operation | open | 182 (61.1) | 97 (64.2) | 0.427 | 0.514 |
|  | endoscope | 116 (38.9) | 54 (35.8) |  |  |
| Scope of surgery | unilateral | 111 (37.2) | 45 (29.8) | 3.053 | 0.217 |
|  | bilateral | 42 (14.1) | 28 (18.5) |  |  |
|  | thyroidectomy and lymphatic dissection | 145 (48.7) | 78 (51.7) |  |  |
| Levothyroxine | yes | 294 (98.7) | 148 (98.0) | 0.271 | 0.603 |
|  | no | 4 (1.3) | 3 (2.0) |  |  |
| Daily activities/days | 0 | 49 (16.4) | 19 (12.6) | 3.075 | 0.38 |
|  | 1-2 | 118 (39.6) | 62 (41.1) |  |  |
|  | 3-4 | 75 (25.2) | 33 (21.9) |  |  |
|  | ≥5 | 56 (18.8) | 37 (24.5) |  |  |
| Daily fruit and vegetable intake (portions^#^) | <1 | 60 (20.1) | 28 (18.5) | 0.174 | 0.917 |
|  | 1-2 | 208 (69.8) | 108 (71.5) |  |  |
|  | 3-4 | 30 (10.1) | 15 (9.9) |  |  |

^a^ Fisher’s exact test was used.

^#^ Fruit and vegetable intake was evaluated with a separate item asking for the number of servings of fruit and vegetable consumed per day (including examples of servings).

Bold values indicate statistical significance (^*^*P* <0.05; ^**^*P* <0.01; ^***^*P* <0.001).

# Supplementary Figures

**Supplementary Figure 1**

**Supplementary Figure 2**


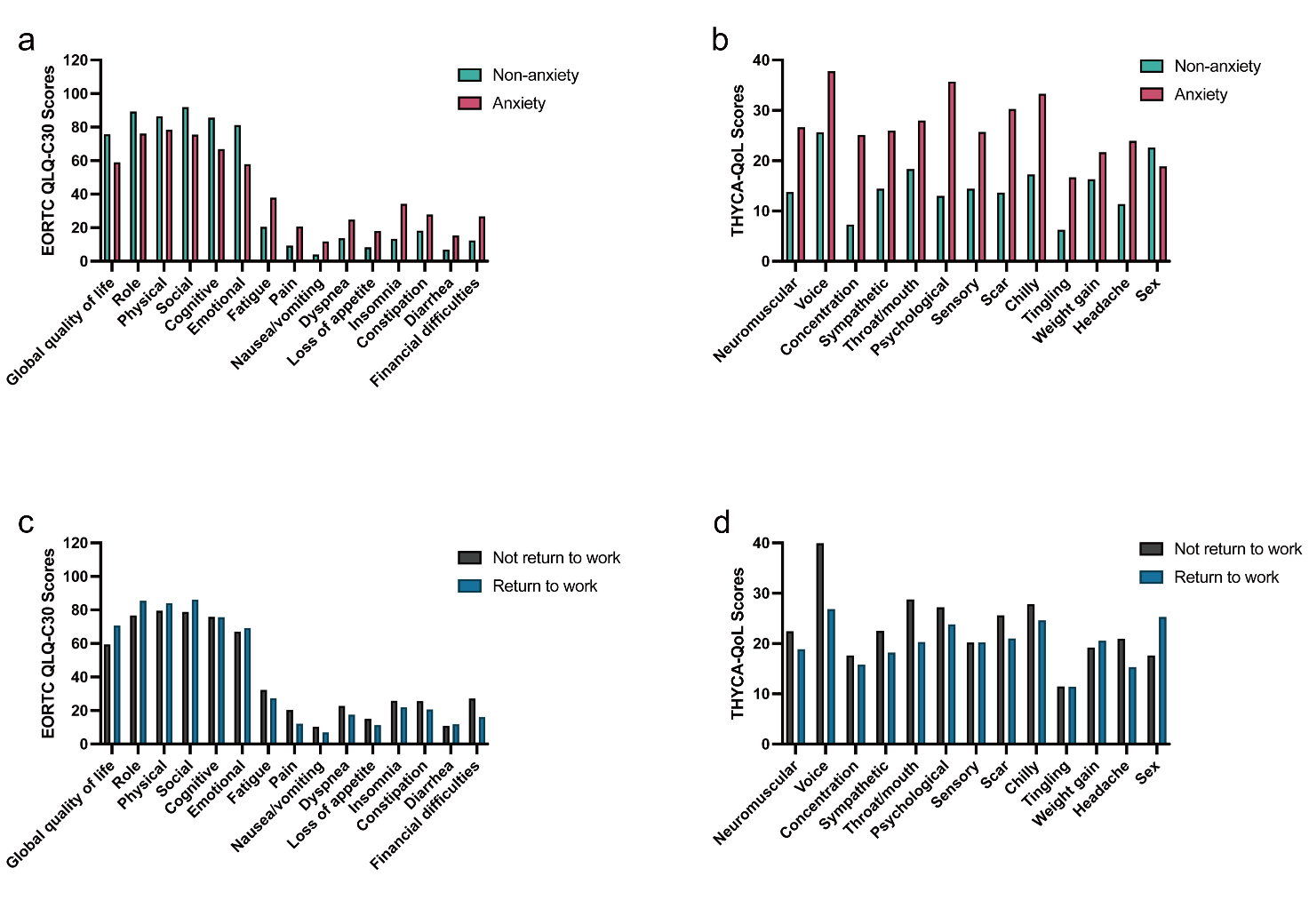


**Figure S2 | Mean scores of EORTC QLQ-C30 and THYCA-QoL in the study population.**

(a). Mean scores of all scales of EORTC QLQ-C30 (including a global QoL scale, five functional scales, e.g., physical, role, cognitive, emotional, and social), three symptom scales, e.g., fatigue, pain, nausea, and vomiting; and several single items assessing common symptoms, e.g., dyspnea, loss of appetite, insomnia, constipation, diarrhea, and financial difficulties) in two groups of patients with different anxious statuses. (b). Mean scores of all scales of THYCA-QoL (including seven symptom scale, e.g., neuromuscular, voice, concentration, sympathetic, throat/mouth, psychological, and sensory; and six single items, e.g. scar, chilly, tingling, weight gain, headache, and sex) in two groups of patients with different anxious statuses. (c). Mean scores of all scales of EORTC QLQ-C30 in two groups of patients with different returning-to-work statuses. (d). Mean scores of all scales of THYCA-QoL in two groups of patients with different returning-to-work statuses.
